# Non-alcoholic Fatty Liver Disease in Patients With Type 2 Diabetes Mellitus: A Cross-Sectional Study Assessing Metabolic Indices and Microvascular Complications

**DOI:** 10.7759/cureus.109099

**Published:** 2026-05-18

**Authors:** Shubham R Desale, Shubhransu Patro, Sambit Das, Varun Jindal, Sai Sri Karlapudi, Mouneesh Karanam, Prithviraj U Naik, Jyoti Prakash Sahoo

**Affiliations:** 1 General Medicine, Kalinga Institute of Medical Sciences, Bhubaneswar, IND; 2 Endocrinology, Kalinga Institute of Medical Sciences, Bhubaneswar, IND; 3 Pharmacology, Kalinga Institute of Medical Sciences, Bhubaneswar, IND

**Keywords:** diabetic nephropathy (dn), diabetic neuropathy (dn), diabetic retinopathy, fatty liver index, fib-4 index, metabolic score for insulin resistance (mets-ir), metabolic syndrome, nafld fibrosis score (nfs), nonalcoholic fatty liver disease (nafld), type 2 diabetes mellitus (type 2 dm)

## Abstract

Background and objectives

Liver fibrosis is a key stage in chronic liver disease progression, marked by excessive extracellular matrix (ECM) deposition that disrupts liver architecture. Non-alcoholic fatty liver disease (NAFLD), a hepatic manifestation of metabolic syndrome, is a growing global health concern. This is especially true for individuals with type 2 diabetes mellitus (T2DM). Chronic hyperglycemia in T2DM may accelerate hepatic fibrogenesis. This happens through oxidative stress, inflammation, and impaired lipid metabolism. We aimed to determine the prevalence of NAFLD, hypertension, and microvascular complications among individuals with T2DM. We assessed their clinical parameters, such as glycemic, lipid, renal, and hepatic values. We also computed and compared the following metabolic indices among patients with and without NAFLD: NAFLD fibrosis score (NFS), fibrosis-4 (FIB4) index, fatty liver index (FLI), and metabolic score for insulin resistance (METS-IR). We further assessed correlations among various clinical parameters and metabolic indices. In addition, we calculated sensitivity, specificity, diagnostic accuracy, and the area under the curve (AUC) for these metabolic indices through a receiver operating characteristic (ROC) curve.

Methods

This cross-sectional study took place at Kalinga Institute of Medical Sciences (KIMS), Bhubaneswar, India, from March 2023 to February 2025. We reviewed adult diabetic patients of both sexes attending our medicine and endocrinology outpatient departments (OPDs) during the study period. We assessed patients referred for ultrasonography to rule out NAFLD. We evaluated clinical parameters and metabolic indices in patients with and without NAFLD. Associations were measured with Spearman’s correlation. We calculated the sensitivity, specificity, AUC, and diagnostic accuracy for these indices. R software (version 4.5.3; R Foundation for Statistical Computing, Vienna, Austria) was used for data analysis.

Results

Among 401 patients with diabetes (median age 68.0 years), 166 (41.4%) were male. NAFLD was present in 115 (28.7%), and hypertension in 183 (45.6%). Neuropathy, nephropathy, and retinopathy affected 147 (36.7%), 142 (35.4%), and 102 (25.4%) of patients, respectively; these rates were higher in those with NAFLD. Patients with NAFLD had higher median glycated hemoglobin (9.11% vs. 8.61%, p < 0.001), METS-IR (48.22 vs. 47.21, p = 0.024), FLI (66.20 vs. 54.55, p < 0.001), FIB4 (1.02 vs. 0.85, p = 0.010), and NFS (1.39 vs. 1.06, p = 0.002) compared to those without NAFLD. Strong positive correlations were observed between FLI and METS-IR (r = 0.88) and NFS and FIB4 (r = 0.87). FLI and METS-IR showed higher specificity (0.8252 and 0.7630) and accuracy (0.7481 and 0.6434), while FIB4 and NFS had moderate sensitivity and diagnostic accuracy. All indices had similar AUC values (range: 0.5723-0.6222).

Conclusion

The study found that chronic hyperglycemia is closely associated with increased liver fibrosis, as reflected in higher metabolic index values among patients with NAFLD. Metabolic indices such as FLI, METS-IR, FIB4, and NFS showed moderate utility as non-invasive markers for glycemic control and fibrosis risk. Incorporating fibrosis assessment into routine diabetes management could enable earlier detection and intervention. The findings underscore the importance of further longitudinal studies to clarify whether improved glycemic control can reverse liver fibrosis.

## Introduction

The rise in type 2 diabetes mellitus (T2DM) and obesity has led to increased prevalence of non-alcoholic fatty liver disease (NAFLD) [1,2]. NAFLD covers liver conditions such as steatosis, non-alcoholic steatohepatitis (NASH), advanced fibrosis, and cirrhosis [3,4]. The global prevalence of NAFLD is 23-25% [1,2]. Increased extracellular matrix (ECM) protein in mesenchymal stromal cells is a hallmark of NAFLD and fibrosis [3-5].

T2DM, insulin resistance, and obesity are major risk factors for NAFLD and advanced fibrosis [5,6]. High blood sugar leads to increased oxidative stress, impaired mitochondrial function, and fat accumulation in liver cells [3-5,7]. These changes activate liver stellate cells, which form scar tissue and ECM proteins [4,8]. Also, metabolic abnormalities in diabetes worsen liver damage and increase the risk of fibrosis [5,6,9]. Studies show a bidirectional relationship between hypertension and NAFLD regardless of other factors [10,11]. Liver fat in NAFLD increases insulin resistance, raising the risk of microvascular complications (neuropathy, nephropathy, and retinopathy) [12,13].

Ultrasonography (USG) is routinely used to assess fatty liver disease [14]. Diagnosing liver fibrosis depends on liver biopsy, but this invasive method has limited inter-rater reliability and risks sampling error [15]. Recently, non-invasive alternatives have emerged, including the NAFLD fibrosis score (NFS) [16], fibrosis-4 (FIB4) index [17], fatty liver index (FLI) [18], and metabolic score for insulin resistance (METS-IR) [19]. These indices offer cost-effective substitutes for USG and biopsy, using standard clinical and laboratory data to help clinicians stratify fibrosis risk with reasonable accuracy [16-19].

We aimed to measure prevalence rates of NAFLD, hypertension, and microvascular complications in individuals with T2DM. We compared the following clinical metrics of patients with and without NAFLD: fasting blood sugar (FBS), two-hour postprandial blood sugar (PPBS), glycated hemoglobin (HbA_1c_), serum creatinine, estimated glomerular filtration rate (eGFR), serum total cholesterol, triglycerides, high-density lipoproteins (HDL), aspartate transaminase (AST), alanine transaminase (ALT), gamma-glutamyl transferase (GGT), serum albumin, and total platelet count. We calculated and compared NFS, FIB4, FLI, and METS-IR scores for both groups. We also assessed correlations among clinical and metabolic data, and calculated sensitivity, specificity, diagnostic accuracy, area under the curve (AUC), and thresholds for these indices.

## Materials and methods

We conducted this cross-sectional study at KIMS, Bhubaneswar, India, from March 2023 to February 2025. The Institutional Ethics Committee (KIIT/KIMS/IEC/1170/2023, dated 27.02.2023) granted us ethics approval before the commencement of the study.

Study criteria

We reviewed adult patients with diabetes who visited our medicine and endocrinology outpatient clinics during the study period. Included patients had routine physician-ordered tests: glycemic parameters (FBS, PPBS, and HbA_1c_), liver function tests (AST, ALT, GGT, and albumin), kidney function tests (serum creatinine and eGFR), lipids (serum total cholesterol, triglycerides, and HDL), complete blood count (CBC), and abdominal USG. We excluded those with autoimmune disease, immunocompromised status, cancer, recent blood transfusion, current use of anticancer drugs, steroids, or antibiotics. We also excluded patients with other chronic liver diseases such as viral hepatitis, autoimmune liver disease, or drug-induced injury, as well as those with cancer, pregnancy, or systemic illnesses affecting the liver.

Study procedure

Data collection involved the evaluation of demographic, clinical, and biochemical parameters. Demographic information included age, gender, height, weight, body mass index (BMI), waist circumference, and marital status, while central obesity was assessed separately using waist circumference. We obtained a detailed medical history for each participant, focusing on hypertension and microvascular complications (neuropathy, nephropathy, and retinopathy), and reviewed previous prescriptions and reports for further evidence of microvascular complications. We then analyzed the distribution of the study population by gender, NAFLD, microvascular complications, and hypertension. The following tests were performed: FBS, PPBS, HbA_1c_, serum creatinine, eGFR, serum total cholesterol, triglyceride, HDL, AST, ALT, GGT, serum albumin, and total platelet count. Next, we computed metabolic indices (NFS, FIB4, FLI, and METS-IR) and compared them between patients with and without NAFLD. We further analyzed correlations between clinical parameters and metabolic indices, and plotted receiver operating characteristic (ROC) curves for the metabolic indices to determine their AUCs and 95% confidence intervals (CIs).

Study tools

We employed four metabolic indices (i.e., NFS, FIB4, FLI, and METS-IR) in this study to assess NAFLD and liver fibrosis. These are non-invasive serum-based indices computed from various clinical and laboratory parameters.

NFS: This index identifies the presence and severity of liver fibrosis in patients with NAFLD [16]. It is a non-invasive, less expensive option than liver biopsy for prognosticating hepatic fibrosis. It is calculated using the following six parameters: age, BMI, diabetes status, serum AST-to-ALT ratio, total platelet count, and serum albumin. The formula is as follows [16]:

NFS = -1.675 + 0.037 x age (years) + 0.094 x BMI (kg/m^2^) + 1.13 (impaired fasting glycemia/diabetes (yes = 1, no = 0)) + 0.99 x AST/ALT ratio - 0.013 x platelet count (10^9^/L) - 0.66 x serum albumin (g/dL).

Angulo et al. [16] found that NFS > 0.676 indicated significant fibrosis.

FIB4: Sterling et al. [17] developed this model to prognosticate liver fibrosis in patients with HIV-HCV coinfection. It is computed using the following four parameters: age, total platelet count, serum AST, and serum ALT. The formula is as follows [17]:

FIB4 = age (years) x AST (U/L)/platelet count (10^9^/L) x AST (U/L)^1/2^.

They found that FIB4 < 1.45 ruled out advanced fibrosis with 70% sensitivity [17].

FLI: Bedogni et al. [18] developed this model to evaluate the risk of fatty liver among patients referred for USG and counseled for lifestyle modification. Its calculation is based on the following four parameters: BMI, waist circumference, GGT, and serum triglyceride. The formula is as follows [18]:

FLI = e^y^/(1+e^y^) x 100, where y = 0.953 x ln (serum triglyceride (mg/dL) + 0.139 x BMI (kg/m^2^) + 0.718 x ln (GGT (U/L) + 0.053 x waist circumference (cm) - 15.745, and ln is the natural logarithm.

They found that an FLI ≥ 60 ruled in hepatic steatosis, as detected by USG [18]. The sensitivity and specificity were 61% and 86%, respectively.

METS-IR: Bello-Chavolla et al. [19] developed this model using anthropometric measurements and non-insulin-fasting laboratory parameters to evaluate insulin sensitivity and resistance. Insulin resistance is an important risk factor for NAFLD and progression of NASH [9]. METS-IR uses glucose levels, adiposity, and lipid profile to assess NAFLD risk without requiring serum insulin levels [20]. It is calculated using the following four parameters: BMI, FBS, HDL, and serum triglycerides. The formula is as follows [19]:

METS-IR = ln (2 x FBS (mg/dL) + serum triglyceride (mg/dL) x BMI (kg/m^2^)/ln (HDL (mg/dL), where ln is the natural logarithm.

Bello-Chavolla et al. [19] found that METS-IR > 51.13 detected insulin resistance with 85% sensitivity and 75% specificity. Zhao et al. [20] found that patients with METS-IR > 39.12 had a 1.06-fold increased risk of developing NAFLD.

Statistical analysis

Non-probability consecutive sampling was used for this cross-sectional study. The Kolmogorov-Smirnov test was used to assess the normality of data distribution. The continuous variables are depicted as medians and interquartile ranges (IQRs). The categorical variables are depicted as frequencies and proportions. The continuous and categorical variables were analyzed using the Wilcoxon rank-sum test and the chi-square test, respectively. We used Spearman’s correlation to assess the association between various parameters. The ROC curve analyses were performed for the four metabolic indices. The AUCs and their 95% CIs were computed. Version 4.5.3 of the R software (R Foundation for Statistical Computing, Vienna, Austria, https://www.R-project.org/) was leveraged for data analysis and generation of plots [21]. A p-value ≤ 0.05 was considered statistically significant.

## Results

**Figure 1:**
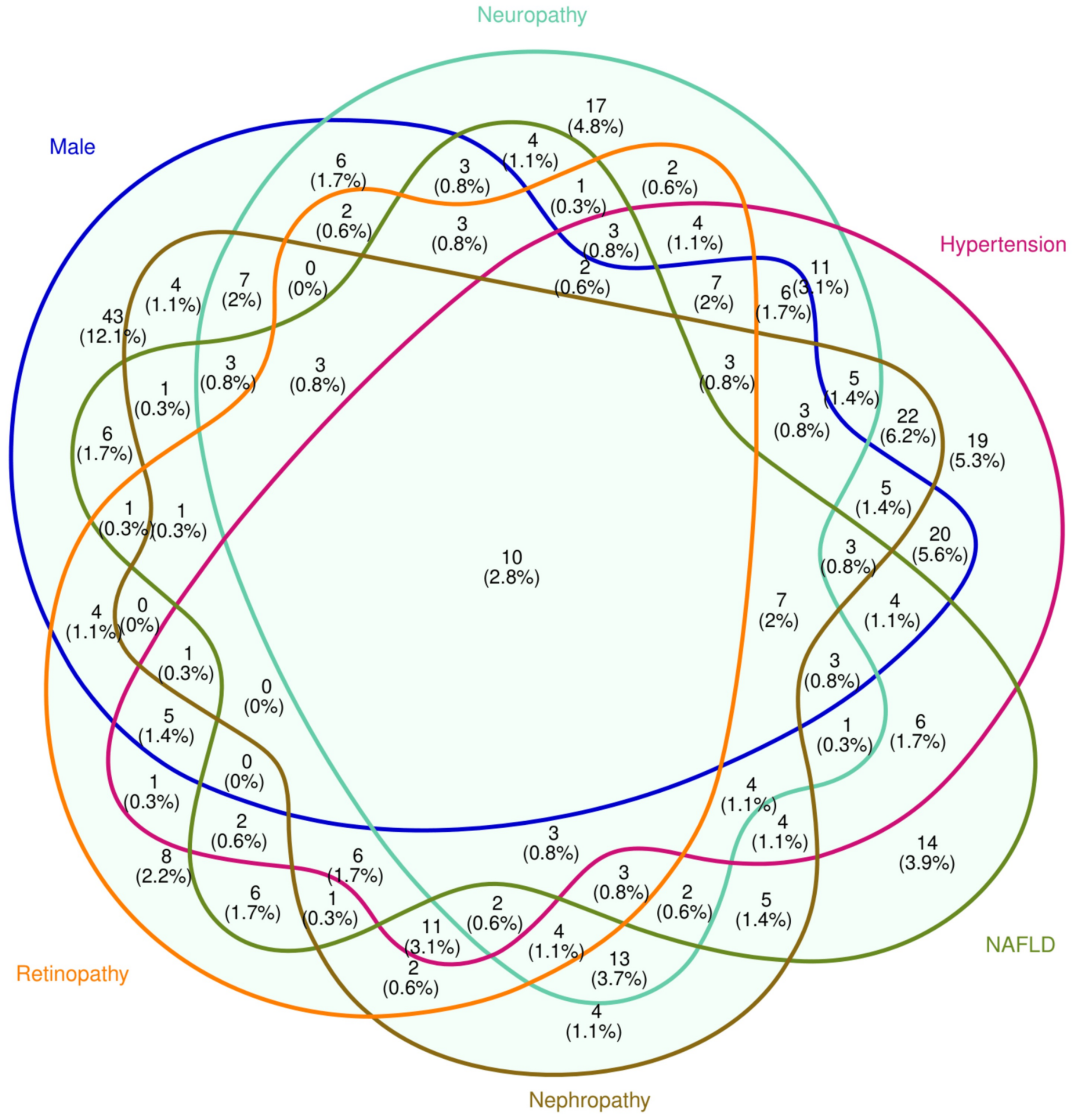
Distribution of study participants The Venn diagram illustrates the number of male participants with NAFLD, hypertension, and diabetic microvascular complications (i.e., neuropathy, nephropathy, and retinopathy). R software (version 4.5.3; R Foundation for Statistical Computing, Vienna, Austria, https://www.R-project.org/) [21] was used to generate this plot. NAFLD: non-alcoholic fatty liver disease

During the study period, 3,217 patients with diabetes visited KIMS. Twenty-five hundred eighty-three (80.3%) subjects were advised for all the following routine tests: glycemic parameters, LFT, KFT, fasting lipid profile, CBC, and USG. Eight hundred seventy (27.0%) subjects declined to participate. Thirteen hundred twelve (40.8%) subjects did not undergo all the required clinical tests. The remaining 401 (12.5%) subjects were assessed in this study. The Venn diagram in Figure 1 portrays the incidences of NAFLD, hypertension, neuropathy, nephropathy, and retinopathy among the male subjects. Table 1 presents the demographic characteristics of the 401 participants. The median age of the study population was 68.0 (58.0-76.0) years. Of them, 166 (41.4%) participants were males. The median BMI was 26.9 kg/m^2,^ 183 (45.6%) subjects were on antihypertensives, and 115 (28.7%) subjects had NAFLD. Diabetic neuropathy, nephropathy, and retinopathy were seen in 147 (36.7%), 142 (35.4%), and 102 (25.4%) subjects, respectively. The incidence of microvascular complications was higher among those with NAFLD.

**Table 1:** Demographic traits of study participants (n = 401) Continuous data are reported as medians and IQRs. Categorical data are presented as numbers and percentages. The continuous data were analyzed using the Wilcoxon rank-sum test, and W-values were calculated. Categorical data were analyzed using the chi-square test, and chi-square values were calculated. Statistical significance was set at p < 0.05. NAFLD: non-alcoholic fatty liver disease; BMI: body mass index; IQR: interquartile range

Parameters	Total (n = 401)	NAFLD (n = 115)	No NAFLD (n = 286)	Statistical test used	Test statistics	p-value
Age (years)	68.0 (58.0-76.0)	69.0 (63.0-80.0)	67.0 (56.0-75.0)	Wilcoxon test	19969	< 0.001
Age > 65 years	234 (58.4%)	74 (64.3%)	160 (55.9%)	Chi-square test	27.08	< 0.001
Gender						
Male	166 (41.4%)	50 (43.5%)	116 (40.6%)	Chi-square test	94.36	< 0.001
Female	235 (58.6%)	65 (56.5%)	170 (59.4%)			
Marital status						
Unmarried	18 (4.5%)	7 (6.5%)	11 (3.8%)	Chi-square test	210.39	< 0.001
Married	349 (87.0%)	85 (78.7%)	264 (90.1%)			
Widowed/divorced	34 (8.5%)	16 (14.8%)	18 (6.1%)			
BMI (kg/m^2^)	26.9 (25.9-28.0)	27.5 (25.7-28.9)	26.8 (25.9-27.6)	Wilcoxon test	19759	0.002
Waist circumference (cm)	89.6 (85.5-93.8)	93.0 (85.4-97.6)	89.5 (85.5-93.2)	Wilcoxon test	19779	0.002
Hypertension	183 (45.6%)	58 (50.4%)	125 (43.7%)	Chi-square test	24.82	< 0.001
Neuropathy	147 (36.7%)	55 (47.8%)	92 (32.2%)	Chi-square test	31.97	< 0.001
Nephropathy	142 (35.4%)	56 (48.7%)	86 (30.1%)	Chi-square test	34.06	< 0.001
Retinopathy	102 (25.4%)	45 (39.1%)	57 (19.9%)	Chi-square test	26.76	< 0.001

**Figure 2:**
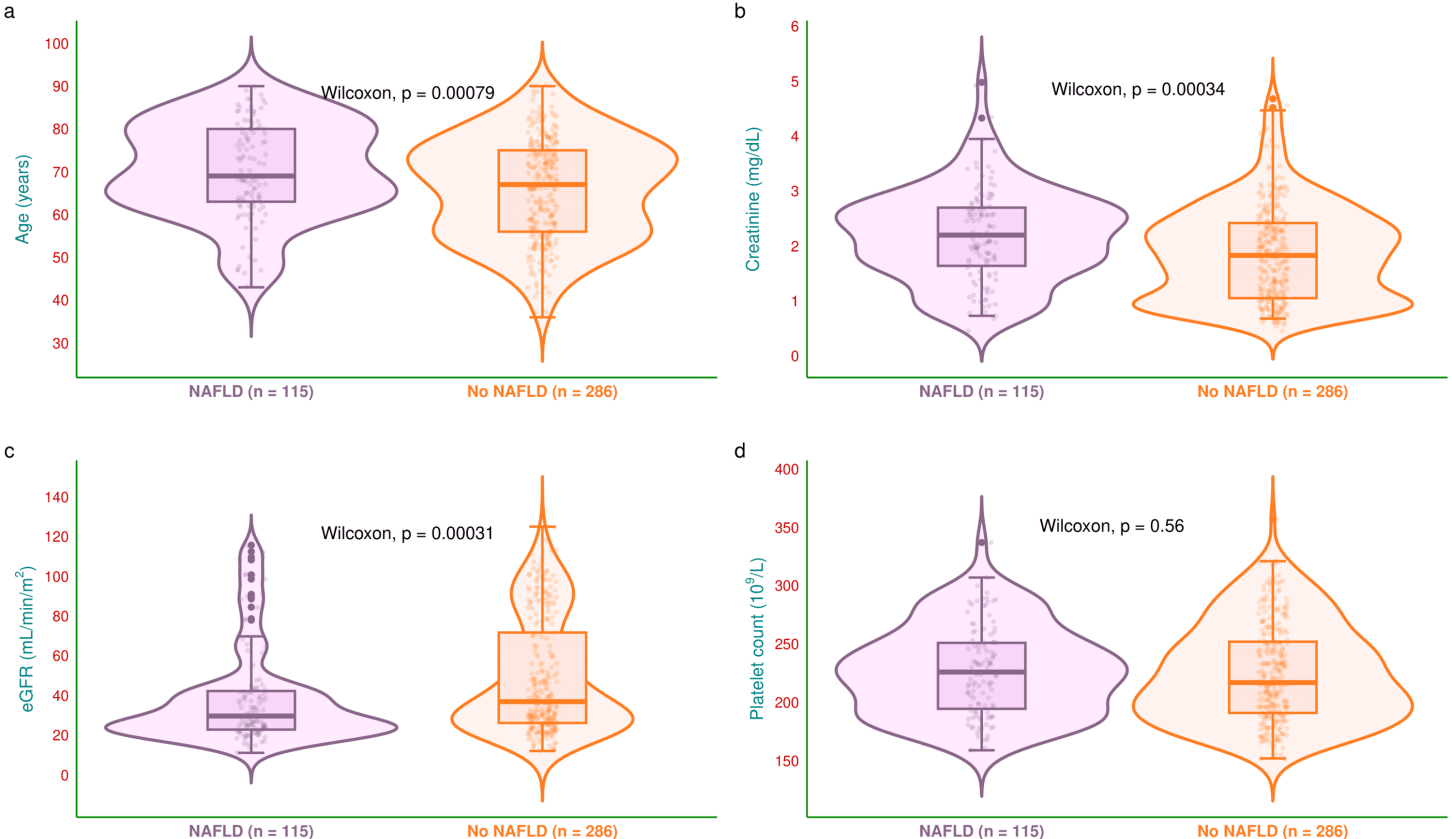
Comparison of age, serum creatinine, eGFR, and total platelet count of participants with and without NAFLD The violin, box-whisker, and jitter plots illustrate the age, serum creatinine, eGFR, and total platelet count of the study participants. The panels a-d show age, serum creatinine, eGFR, and total platelet count of participants with and without NAFLD, respectively. These data were analyzed using the Wilcoxon rank-sum test. Statistical significance was set at p < 0.05. R software (version 4.5.3; R Foundation for Statistical Computing, Vienna, Austria, https://www.R-project.org/) [21] was used to generate this plot. eGFR: estimated glomerular filtration rate; NAFLD: non-alcoholic fatty liver disease

Figure 2 presents age, serum creatinine, eGFR, and total platelet count for study participants with and without NAFLD. The median ages of the subjects with and without NAFLD were 69.0 years and 67.0 years, respectively (Figure 2a). The difference was statistically significant (p < 0.001). The median serum creatinine values of the corresponding groups were 2.20 mg/dL and 1.83 mg/dL, respectively (Figure 2b). The difference was statistically significant (p < 0.001). The median eGFR values were 29.64 mL/min/m^2^ and 36.88 mL/min/m^2^, respectively (Figure 2c). The difference was statistically significant (p < 0.001). The median total platelet counts for the two groups were 226.0 × 10^9^/L and 217 × 10^9^/L, respectively (Figure 2d). The difference was not statistically significant (p = 0.557). Table 2 shows these data and their statistics.

**Table 2:** Clinical parameters of the study participants (n = 401) Continuous data are reported as medians and IQRs. These data were analyzed using the Wilcoxon rank-sum test, and W-values were calculated. Statistical significance was set at p < 0.05. NAFLD: non-alcoholic fatty liver disease; FBS: fasting blood sugar; PPBS: two-hour post-prandial blood sugar; HbA_1c_: glycated hemoglobin; AST: aspartate transaminase; ALT: alanine transaminase; GGT: gamma-glutamyl transferase; HDL: high-density lipoprotein; eGFR: estimated glomerular filtration rate; IQR: interquartile range

Parameters	Total (n = 401)	NAFLD (n = 115)	No NAFLD (n = 286)	W-value	p-value
FBS (mg/dL)	183.0 (152.0-215.0)	185.0 (156.0-215.5)	180.5 (151.0-213.5)	17422	0.352
PPBS (mg/dL)	327.0 (272.0-368.0)	334.0 (282.0-372.5)	325.5 (272.0-367.0)	17809	0.194
HbA_1c_ (%)	8.74 (7.91-9.47)	9.11 (8.21-9.88)	8.61 (7.81-9.23)	20268	< 0.001
AST (IU/L)	169.0 (90.0-247.0)	171.0 (104.0-251.0)	167.5 (86.0-245.8)	17225	0.458
ALT (IU/L)	227.0 (128.0-443.0)	272.0 (147.5-532.0)	219.0 (120.0-414.3)	18157	0.103
GGT (IU/L)	52.0 (48.0-56.0)	52.0 (50.0-56.0)	50.0 (48.0-56.0)	18942	0.017
Serum albumin (g/dL)	2.9 (2.7-3.2)	2.9 (2.7-3.2)	3.0 (2.7-3.2)	15896	0.600
Serum cholesterol (mg/dL)	176.0 (148.0-206.0)	192.0 (162.5-214.0)	167.0 (146.0-202.3)	20674	< 0.001
Serum triglyceride (mg/dL)	216.0 (186.0-244.0)	220.0 (189.0-252.0)	214.5 (185.0-241.8)	17862	0.177
Serum HDL (mg/dL)	37.0 (35.0-40.0)	38.0 (35.0-40.0)	37.0 (34.0-40.0)	17785	0.200
Platelet count (10^9^/L)	221.0 (192.0-251.0)	226.0 (194.5-251.0)	217.0 (191.0-252.0)	17063	0.557
Serum creatinine (mg/dL)	1.94 (1.21-2.50)	2.20 (1.64-2.70)	1.83 (1.05-2.42)	20205	< 0.001
eGFR (mL/min/m^2^)	34.49 (25.03-62.35)	29.64 (22.80-42.25)	36.88 (26.15-71.68)	12662	< 0.001

**Figure 3:**
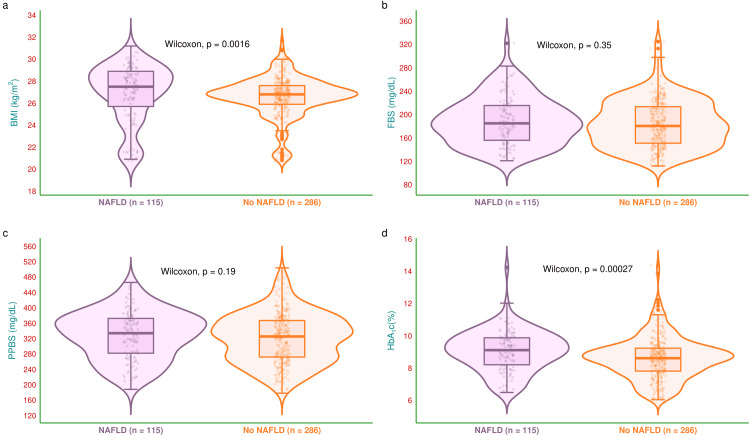
Comparison of BMI and glycemic parameters of participants with and without NAFLD The violin, box-whisker, and jitter plots illustrate the BMI and glycemic parameters of the study participants. The panels a-d show BMI, FBS, PPBS, and HbA_1c_ of participants with and without NAFLD, respectively. These data were analyzed using the Wilcoxon rank-sum test. Statistical significance was set at p < 0.05. R software (version 4.5.3; R Foundation for Statistical Computing, Vienna, Austria, https://www.R-project.org/) [21] was used to generate this plot. BMI: body mass index; FBS: fasting blood sugar; PPBS: two-hour post-prandial blood sugar; HbA_1c_: glycated hemoglobin; NAFLD: non-alcoholic fatty liver disease

Figure 3 presents BMI and glycemic parameters for study participants with and without NAFLD. The median BMI values of the subjects with and without NAFLD were 27.5 kg/m^2^ and 26.8 kg/m^2^, respectively (Figure 3a). The difference was statistically significant (p = 0.002). The median FBS values of the corresponding groups were 185.0 mg/dL and 180.5 mg/dL, respectively (Figure 3b). The difference was not statistically significant (p = 0.352). The median PPBS values were 334.0 mg/dL and 325.5 mg/dL, respectively (Figure 3c). The difference was not statistically significant (p = 0.194). The median HbA_1c_ values of the two groups were 9.11% and 8.61%, respectively (Figure 3d). The difference was statistically significant (p < 0.001). Table 2 shows these data and their statistics.

**Figure 4:**
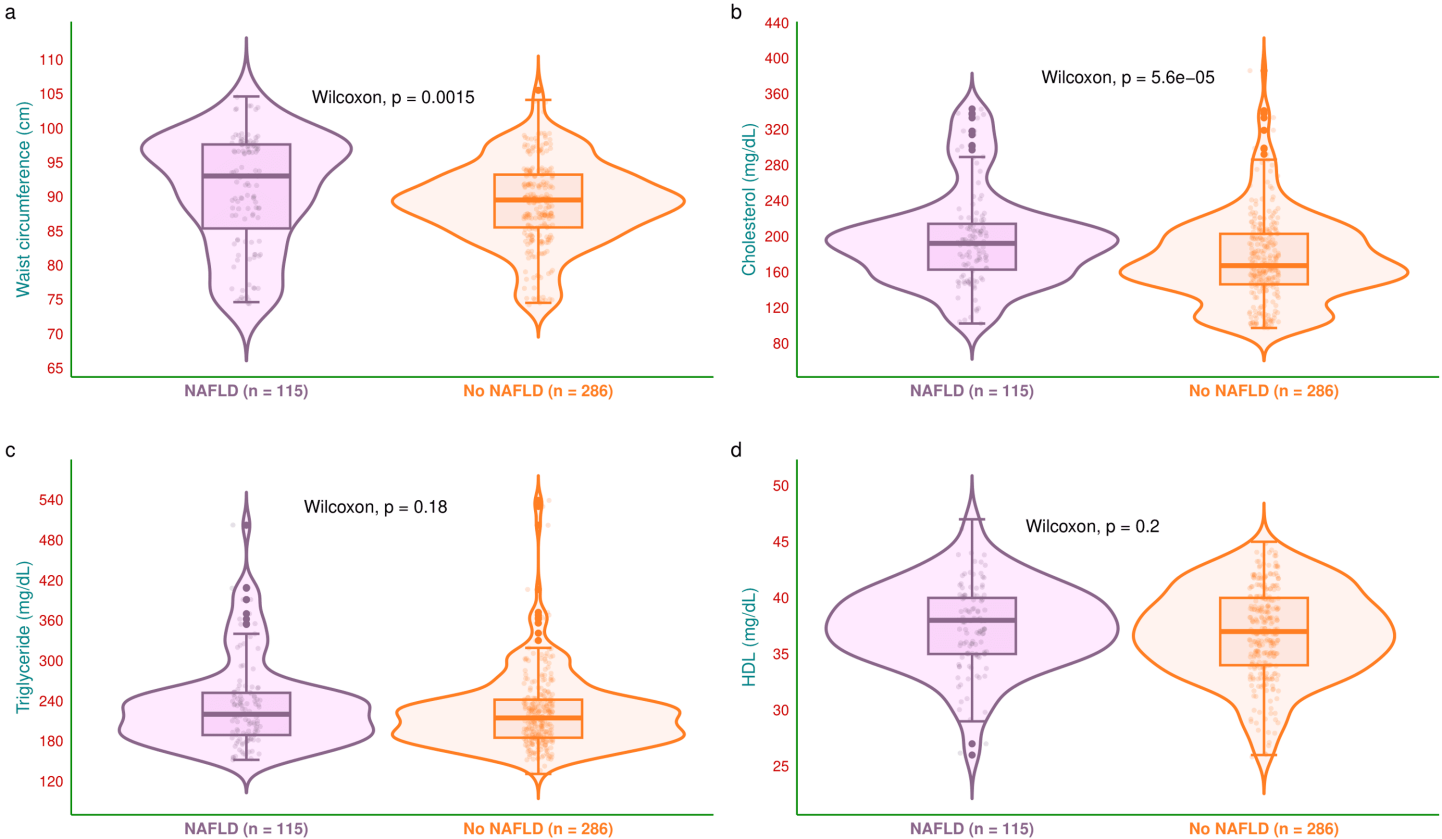
Comparison of waist circumference and lipid profile of participants with and without NAFLD The violin, box-whisker, and jitter plots illustrate the waist circumference and lipid profile of the study participants. The panels a-d show waist circumference, serum total cholesterol, serum triglyceride, and serum HDL of participants with and without NAFLD, respectively. These data were analyzed using the Wilcoxon rank-sum test. Statistical significance was set at p < 0.05. R software (version 4.5.3; R Foundation for Statistical Computing, Vienna, Austria, https://www.R-project.org/) [21] was used to generate this plot. HDL: high-density lipoprotein; NAFLD: non-alcoholic fatty liver disease

Figure 4 presents waist circumference and lipid profile for study participants with and without NAFLD. The median waist circumferences of the subjects with and without NAFLD were 93.0 cm and 89.5 cm, respectively (Figure 4a). The difference was statistically significant (p = 0.002). The median cholesterol values of the corresponding groups were 192.0 mg/dL and 167.0 mg/dL, respectively (Figure 4b). The difference was statistically significant (p < 0.001). The median triglyceride values were 220.0 mg/dL and 214.5 mg/dL, respectively (Figure 4c). The difference was not statistically significant (p = 0.177). The median HDL values of the two groups were 38.0 mg/dL and 37.0 mg/dL, respectively (Figure 4d). The difference was not statistically significant (p = 0.200). Table 2 shows these data and their statistics.

**Figure 5:**
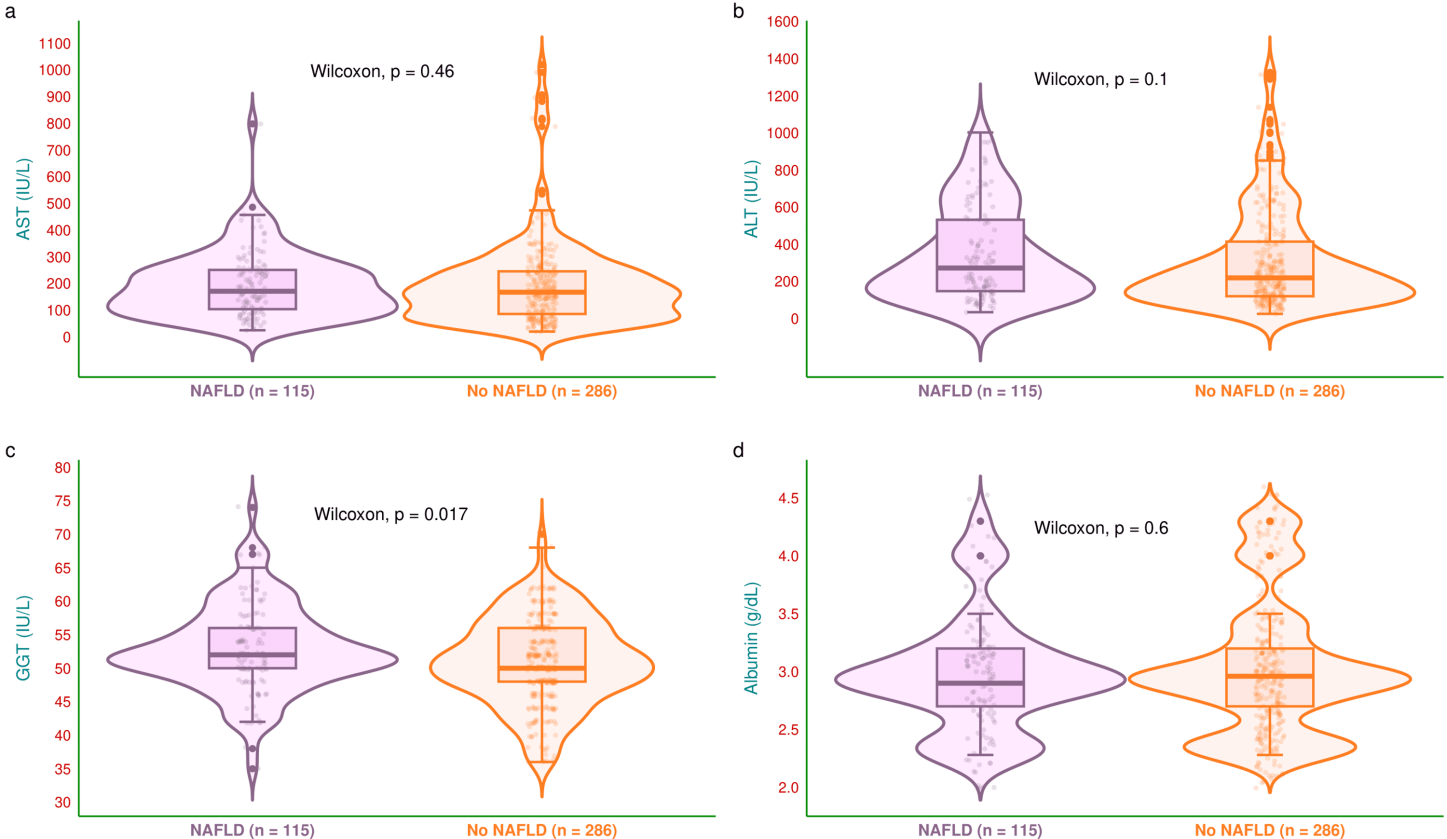
Comparison of liver enzymes and serum albumin of participants with and without NAFLD The violin, box-whisker, and jitter plots illustrate the liver enzymes and serum albumin of the study participants. The panels a-d show AST, ALT, GGT, and serum albumin levels of participants with and without NAFLD, respectively. These data were analyzed using the Wilcoxon rank-sum test. Statistical significance was set at p < 0.05. R software (version 4.5.3; R Foundation for Statistical Computing, Vienna, Austria, https://www.R-project.org/) [21] was used to generate this plot. AST: aspartate transaminase; ALT: alanine transaminase; GGT: gamma-glutamyl transferase; NAFLD: non-alcoholic fatty liver disease

Figure 5 presents liver enzymes and serum albumin for study participants with and without NAFLD. The median AST values of the subjects with and without NAFLD were 171.0 IU/L and 167.5 IU/L, respectively (Figure 5a). The difference was not statistically significant (p = 0.458). The median ALT values of the corresponding groups were 272.0 IU/L and 219.0 IU/L, respectively (Figure 5b). The difference was not statistically significant (p = 0.103). The median GGT values were 52.0 IU/L and 50.0 IU/L, respectively (Figure 5c). The difference was statistically significant (p = 0.017). The median serum albumin values of the two groups were 2.9 g/dL and 3.0 g/dL, respectively (Figure 5d). The difference was not statistically significant (p = 0.600). Table 2 shows these data and their statistics.

**Figure 6:**
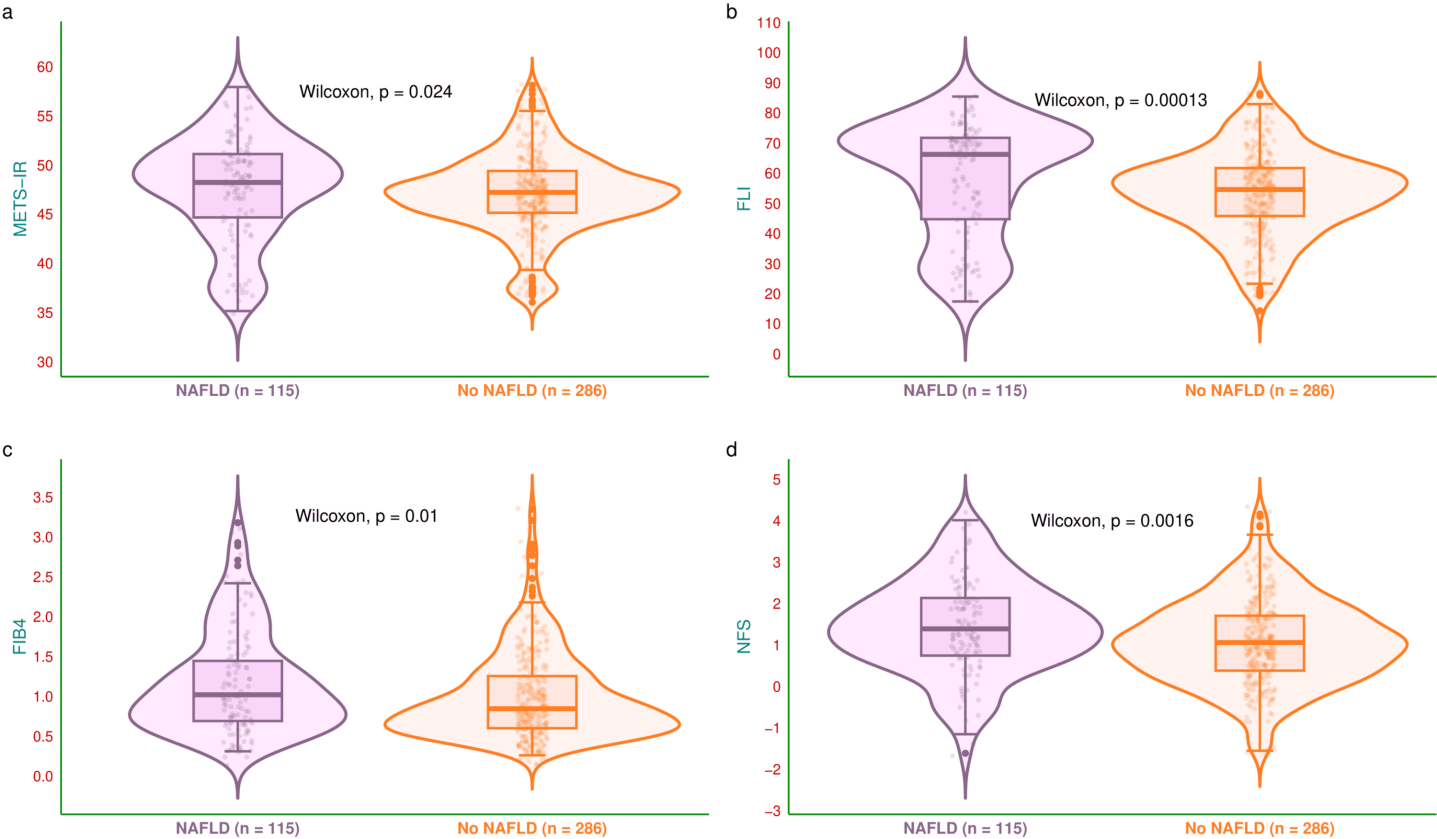
Comparison of metabolic indices of participants with and without NAFLD The violin, box-whisker, and jitter plots illustrate the metabolic indices of the study participants. The panels a-d show the METS-IR, FLI, FIB4, and NFS values of participants with and without NAFLD, respectively. These data were analyzed using the Wilcoxon rank-sum test. Statistical significance was set at p < 0.05. R software (version 4.5.3; R Foundation for Statistical Computing, Vienna, Austria, https://www.R-project.org/) [21] was used to generate this plot. METS-IR: metabolic score for insulin resistance; FLI: fatty liver index; FIB4: fibrosis-4 index; NFS: NAFLD fibrosis score; NAFLD: non-alcoholic fatty liver disease

Figure 6 and Table 3 show metabolic indices of the study participants with and without NAFLD. The median METS-IR values of the subjects with and without NAFLD were 48.22 (44.67-51.11) and 47.21 (45.14-49.39), respectively (Figure 6a). The difference was statistically significant (p = 0.024). The median FLI scores of the corresponding groups were 66.20 (44.70-71.70) and 54.55 (45.78-61.68), respectively (Figure 6b). The difference was statistically significant (p < 0.001). The median FIB4 values were 1.02 (0.69-1.45) and 0.85 (0.60-1.26), respectively (Figure 6c). The difference was statistically significant (p = 0.010). The median NFS values of the two groups were 1.39 (0.75-2.14) and 1.06 (0.38-1.71), respectively (Figure 6d). The difference was statistically significant (p = 0.002).

**Table 3:** Metabolic indices of the study participants (n = 401) Continuous data are reported as medians and IQRs. These data were analyzed using the Wilcoxon rank-sum test, and W-values were calculated. Statistical significance was set at p < 0.05. METS-IR: metabolic score for insulin resistance; FLI: fatty liver index; FIB4: fibrosis-4 index; NFS: NAFLD fibrosis score; NAFLD: non-alcoholic fatty liver disease; IQR: interquartile range

Parameters	Total (n = 401)	NAFLD (n = 115)	No NAFLD (n = 286)	W-value	p-value
METS-IR	47.50 (45.13-50.09)	48.22 (44.67-51.11)	47.21 (45.14-49.39)	18823	0.024
FLI	55.90 (45.40-66.60)	66.20 (44.70-71.70)	54.55 (45.78-61.68)	20465	< 0.001
FIB4	0.87 (0.63-1.32)	1.02 (0.69-1.45)	0.85 (0.60-1.26)	19141	0.010
NFS	1.13 (0.49-1.84)	1.39 (0.75-2.14)	1.06 (0.38-1.71)	19754	0.002

**Figure 7:**
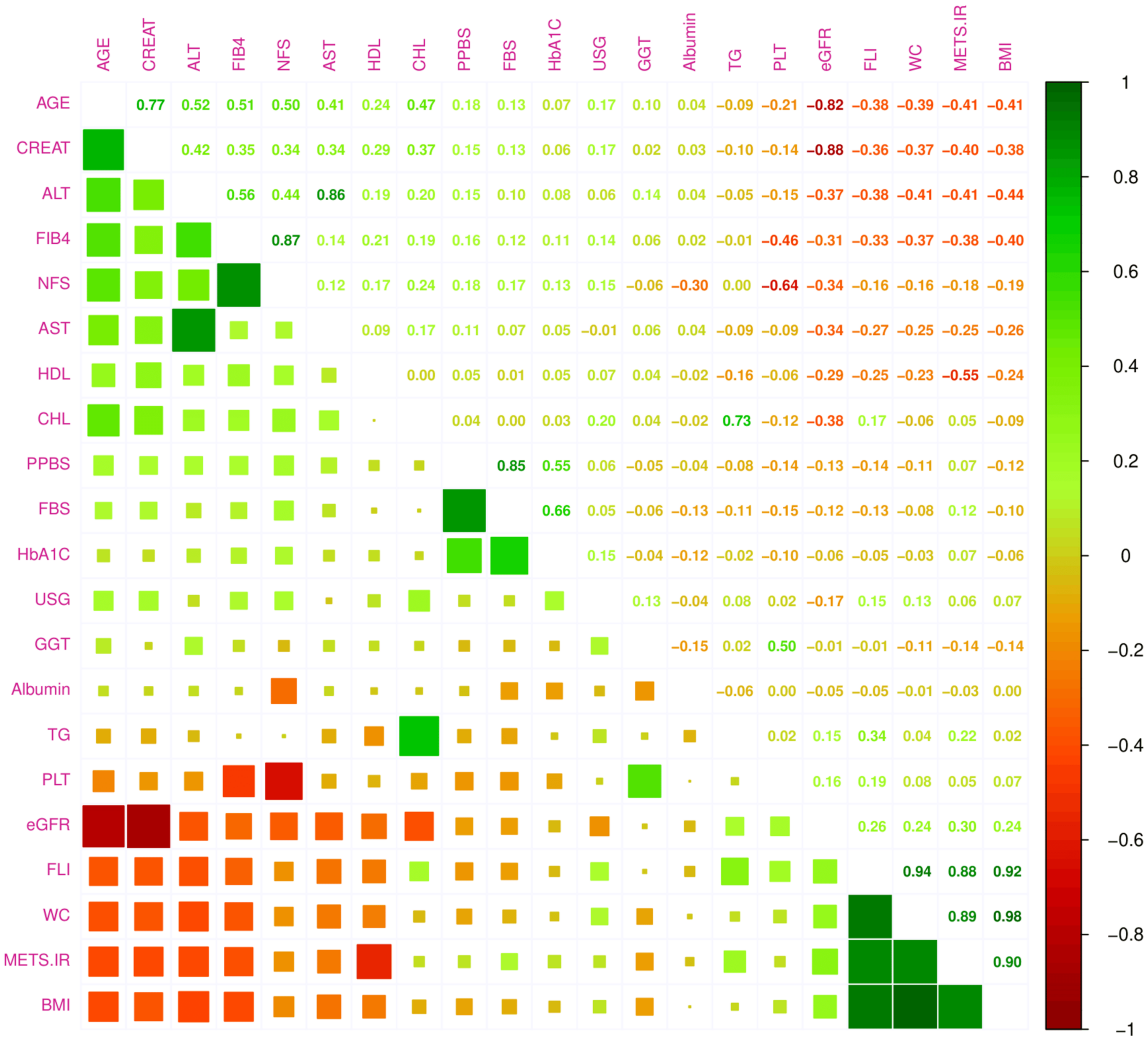
Correlation of various parameters of the study population The correlation plot illustrates the degree of association among various parameters of the study participants. The upper and lower sections show the correlation coefficients and squares (of sizes proportional to the coefficients), respectively. The correlation coefficients range from +1 (strong positive correlation) to -1 (strong negative correlation). R software (version 4.5.3; R Foundation for Statistical Computing, Vienna, Austria, https://www.R-project.org/) [21] was used to generate this plot. CREAT: serum creatinine; ALT: alanine transaminase; FIB4: fibrosis-4 index; NFS: NAFLD fibrosis score; AST: aspartate transaminase; HDL: high-density lipoprotein; CHL: serum total cholesterol; PPBS: two-hour postprandial blood sugar; FBS: fasting blood sugar; HbA_1c_: glycated hemoglobin; USG: ultrasonography; GGT: gamma-glutamyl transferase, TG: serum triglyceride, PLT: total platelet count, eGFR: estimated glomerular filtration rate, FLI: fatty liver index, NAFLD: non-alcoholic fatty liver disease, WC: waist circumference, METS-IR: metabolic score for insulin resistance, BMI: body mass index

Figure 7 illustrates the correlation coefficients among various parameters of the study population. There were positive correlations between BMI and waist circumference (r = 0.98, 95% CI = 0.98 to 0.99, p < 0.001), FLI and waist circumference (r = 0.94, 95% CI = 0.92 to 0.95, p < 0.001), BMI and FLI (r = 0.92, 95% CI = 0.90 to 0.93, p < 0.001), BMI and METS-IR (r = 0.90, 95% CI = 0.88 to 0.92, p < 0.001), METS-IR and waist circumference (r = 0.89, 95% CI = 0.86 to 0.91, p < 0.001), FLI and METS-IR (r = 0.88, 95% CI = 0.86 to 0.90, p < 0.001), NFS and FIB4 (r = 0.87, 95% CI = 0.85 to 0.89, p < 0.001), AST and ALT (r = 0.86, 95% CI = 0.83 to 0.88, p < 0.001), FBS and PPBS (r = 0.85, 95% CI = 0.82 to 0.87, p < 0.001), age and serum creatinine (r = 0.77, 95% CI = 0.73 to 0.81, p < 0.001), and serum cholesterol and triglyceride (r = 0.73, 95% CI = 0.68 to 0.77, p < 0.001). There were negative correlations between eGFR and serum creatinine (r = -0.88, 95% CI = -0.90 to -0.85, p < 0.001), eGFR and age (r = -0.82, 95% CI = -0.85 to -0.79, p < 0.001), and NFS and total platelet count (r = -0.64, 95% CI = -0.70 to -0.58, p < 0.001). We did not find strong associations between fatty liver status and any parameter.

**Figure 8:**
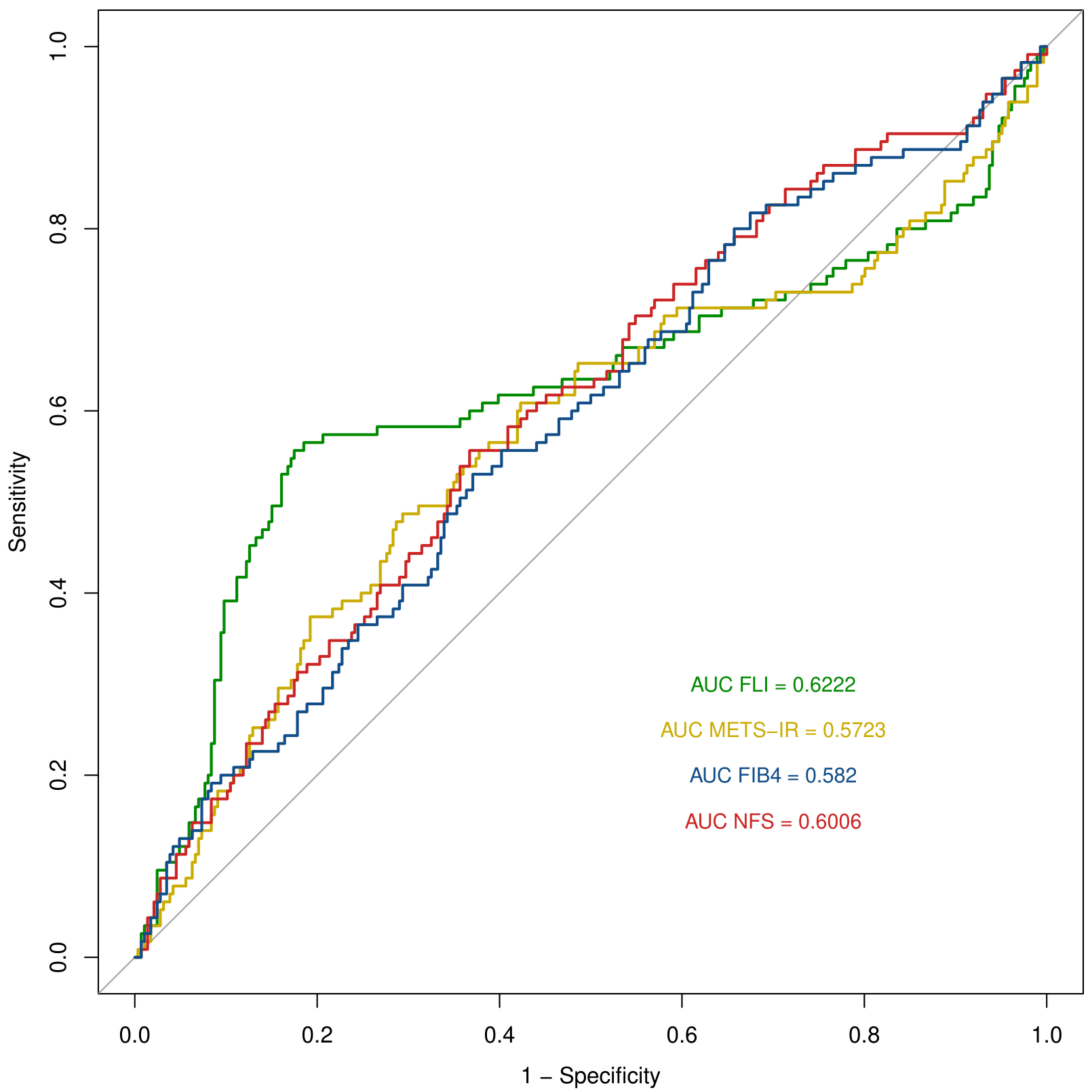
ROC curves for metabolic indices The X- and Y-axes denote 1-specificity and sensitivity values of FLI, METS-IR, FIB4, and NFS. The four ROC curves and their AUC values are illustrated in different colors. R software (version 4.5.3; R Foundation for Statistical Computing, Vienna, Austria, https://www.R-project.org/) [21] was used to generate this plot. FLI: fatty liver index; METS-IR: metabolic score for insulin resistance; FIB4: fibrosis-4 index; NFS: NAFLD fibrosis score; NAFLD: non-alcoholic fatty liver disease; ROC: receiver operating characteristic curve; AUC: area under the curve

Figure 8 illustrates the ROC curves of FLI, METS-IR, FIB4, and NFS. Table 4 presents the sensitivity, specificity, diagnostic accuracy, threshold values, AUCs for ROC curves, and their 95% CIs. The sensitivity values of FLI, METS-IR, FIB4, and NFS were 0.5565, 0.4870, 0.5304, and 0.5478, respectively. The specificity values of FLI, METS-IR, FIB4, and NFS were 0.8252, 0.7630, 0.6189, and 0.6329, respectively. The diagnostic accuracy values of the four indices were 0.7481, 0.6434, 0.5935, and 0.6085, respectively. The threshold values were 64.75, 48.705, 0.975, and 1.34, respectively. The AUCs for FLI, METS-IR, FIB4, and NFS were 0.6222 (0.5519-0.6926), 0.5723 (0.5054-0.6392), 0.5820 (0.5193-0.6447), and 0.6006 (0.5387-0.6625), respectively.

**Table 4:** Statistical comparison of metabolic indices True-positive, false-positive, true-negative, and false-negative values were used to compute the sensitivity, specificity, and diagnostic accuracy of FLI, METS-IR, FIB4, and NFS. The thresholds, AUCs, and 95% CIs for AUC for these indices were calculated from the ROC curves. FLI: fatty liver index; METS-IR: metabolic score for insulin resistance; FIB4: fibrosis-4 index; NFS: NAFLD fibrosis score; NAFLD: non-alcoholic fatty liver disease; ROC: receiver operating characteristic curve; AUC: area under the curve; CI: confidence interval

Parameters	True positive	False positive	True negative	False negative	Sensitivity	Specificity	Diagnostic accuracy	Threshold	AUC	95% CI for AUC
FLI	64	50	51	236	0.5565	0.8252	0.7481	64.75	0.6222	0.5519-0.6926
METS-IR	56	84	59	202	0.4870	0.7063	0.6434	48.705	0.5723	0.5054-0.6392
FIB4	61	109	54	177	0.5304	0.6189	0.5935	0.975	0.5820	0.5193-0.6447
NFS	63	105	52	181	0.5478	0.6329	0.6085	1.34	0.6006	0.5387-0.6625

## Discussion

This study underscores the critical roles of chronic hyperglycemia and microvascular complications in the progression of liver fibrosis, providing evidence for a direct association between T2DM and NAFLD. The elevated NFS, FIB4, FLI, and METS-IR scores among patients with NAFLD suggested an increased risk of hepatic fibrosis in this population. Of the 401 patients with diabetes, 115 (28.7%) had NAFLD. The prevalence data match those of the studies by Mitrovic et al. [1] and Stefan et al. [2]. We found statistically significant differences for the following parameters: age, serum creatinine, eGFR, BMI, HbA_1c_, waist circumference, serum total cholesterol, and GGT. These findings were consistent with those of Zhao et al. [20]. The metabolic indices differed significantly between patients with and without NAFLD. Our data regarding FLI and METS-IR concorded with those of the study by Zou et al. [22]. We found strong positive correlations between NFS and FIB4 (r = 0.87, 95% CI = 0.85 to 0.89, p < 0.001) and FLI and METS-IR (r = 0.88, 95% CI = 0.86 to 0.90, p < 0.001). FLI had the highest diagnostic accuracy (75%), followed by METS-IR (64%), NFS (61%), and FIB4 (59%).

The focal point of systemic insulin resistance is adipose tissue, which serves as a free fatty acid (FFA) pump [9]. Local hypoxia, macrophage infiltration, and fibrosis are triggered by adipocyte hypertrophy in the early phases of obesity. Production of tumor necrosis factor-alpha (TNF-α) and interleukin-6 (IL-6) further intensifies insulin resistance, accelerates lipolysis, and increases FFA efflux [23,24]. Ectopic lipid deposition in the skeletal muscle diminishes glucose uptake and glycogen synthesis, thereby rerouting energy toward hepatic lipogenesis [9,24]. In addition to insulin resistance in skeletal muscle and adipose tissue, hepatic insulin resistance also exists independently [9,23,24]. In individuals with diabetes and NAFLD, hepatic lipid accumulation suppresses the phosphorylation of insulin receptor substrate-1 (IRS-1) and activates protein kinase C (PKC), which can limit the insulin receptor activity and promote lipid accumulation [12,13]. Atherogenic lipoproteinemia and dysglycemia are primarily caused by systemic and hepatic insulin resistance. In patients with T2DM and NAFLD, the risk of cardiovascular disease (CVD) is mediated by both dysglycemia and atherogenic dyslipidemia [12]. In patients with NAFLD, chronic hyperglycemia and insulin resistance facilitate the progression of diabetic neuropathy, nephropathy, and retinopathy [13].

METS-IR integrates three different metabolic dimensions (i.e., adiposity, dyslipidemia, and dysglycemia) to address insulin resistance [20]. Furthermore, the predictability of NAFLD risk has been demonstrated across different regions of the world [25]. A recent meta-analysis by Zhang et al. [26] found that higher NFS and FIB4 scores are associated with an increased risk of CVD. NFS [16] and FIB4 [17] employ age, AST, and ALT, whereas FLI [18] and METS-IR [19] utilize BMI and serum triglycerides for their calculations. These factors could be attributed to the strong positive correlations among NFS and FIB4, and FLI and METS-IR. These four indices had similar sensitivity, specificity, and AUC values in our study.

Strengths and limitations

Our study was strengthened by the analysis of four metabolic indices, their correlations with other parameters, and ROC plots. Despite these significant insights, the study had a few limitations. First, the cross-sectional design precludes causal inferences regarding the relationship between hyperglycemia and fibrosis progression. Second, non-invasive indices, such as NFS, FIB4, FLI, and METS-IR, may lack the precision of histopathological validation via liver biopsy. Third, we were unable to perform predictive modeling of these indices in our study. Future studies should incorporate longitudinal study designs and biopsy data to validate these findings and explore the potential reversibility of fibrosis through improved glycemic control.

## Conclusions

Our findings emphasize the critical need to integrate routine fibrosis risk assessment into standard diabetes care guidelines. Non-invasive, serum-based metabolic indices, such as NFS, FIB4, FLI, and METS-IR, offer cost-effective, accessible tools for identifying high-risk patients with NAFLD, NASH, hepatic fibrosis, and CVD. By leveraging non-invasive scoring methods alongside traditional diabetes management strategies, healthcare providers can facilitate earlier intervention and improve patient outcomes for dysglycemia, insulin resistance, dyslipidemia, adiposity, hepatic steatosis, and fibrosis. Furthermore, these results highlight the interconnected nature of metabolic dysfunction in T2DM, underscoring the importance of interdisciplinary approaches to care. We recommend prospective studies to generalize our study findings through liver biopsy and a sustained euglycemic state.
